# Metformin inhibits tumor growth by regulating multiple miRNAs in human cholangiocarcinoma

**DOI:** 10.18632/oncotarget.3063

**Published:** 2014-12-18

**Authors:** Xingming Jiang, Ning Ma, Dayong Wang, Fuyuan Li, Rongzhang He, Dongliang Li, Ruiqi Zhao, Qingxin Zhou, Yimin Wang, Fumin Zhang, Ming Wan, Pengcheng Kang, Xu Gao, Yunfu Cui

**Affiliations:** ^1^ Department of Hepatopancreatobiliary Surgery, Second Affiliated Hospital of Harbin Medical University, Harbin, China; ^2^ Department of Biochemistry and Molecular Biology, Harbin Medical University, Harbin, China; ^3^ Center for Endemic Disease Control, Harbin Medical University, Harbin, China

**Keywords:** metformin, cholangiocarcinoma, microRNA, cell cycle, prognosis

## Abstract

The antidiabetic drug metformin exerts antineoplastic effects in many types of malignancies, however the effect of metformin on cholangiocarcinoma (CCA) still remains unclear. In the present study, we investigated that metformin treatment was closely associated with the clinicopathologic characteristics and improved postoperative survival of CCA patients. Metformin inhibited CCA tumor growth by cell cycle arrest *in vitro* and *in vivo*. We explored that the expression of six miRNAs (mir124, 182, 27b, let7b, 221 and 181a), which could directly target cell-cycle-regulatory genes, was altered by metformin *in vitro* and *in vivo*. These miRNAs were dysregulated in cholangiocarcinoma and promoted the CCA genesis and metformin exactly modulated these carcinogenic miRNAs expression to arrest the cell cycle and inhibit the proliferation. Meanwhile, these miRNAs expression changes correlated with the tumor volume and postoperative survival of CCA patients and could be used to predict the prognosis. Further we confirmed that metformin upregulated Drosha to modulate these miRNAs expression. Our results elucidated that metformin inhibited CCA tumor growth via the regulation of Drosha-mediated multiple carcinogenic miRNAs expression and comprehensive evaluation of these miRNAs expression could be more efficient to predict the prognosis. Moreover, metformin might be a quite promising strategy for CCA prevention and treatment.

## INTRODUCTION

Cholangiocarcinoma (CCA), the second most common primary hepatobiliary malignancy, is a fatal cancer originating from the neoplastic transformation of biliary epithelial cells and characterized by increasing incidence worldwide and poor prognosis [1]. CCA is most often diagnosed at an advanced stage with intrahepatic and lymph node metastases because the early stages of CCA progression are usually asymptomatic without effective preventive measures [2, 3]. According to clinical statistical study, approximately 7500 new cases of cholangiocarcinoma are diagnosed per year in the United States with less than 30% 5-year survival [4]. Because of resistance to common chemotherapies, up to now the radical surgical resection is still the most powerful therapy for CCA [5].

Metformin is the most widely used antidiabetic drug in the world, introduced into clinical practice in the 1950s for the treatment of type II diabetes [6], and there is increasing evidence of the potential efficacy of this agent as an anticancer drug recently [7, 8]. According to the epidemiologic survey, metformin has significant effects on tumorigenesis in metformin treated patients [9, 10]. It has been reported that patients with type II diabetes who are prescribed metformin have a lower risk of pancreatic cancer than those not taking metformin [11]. In addition, several reports also outlined the decreased cancer incidence and cancer related mortality in the tumor patients who received metformin [10]. Further investigations found that metformin inhibited the proliferation of various human cancer cell types including prostate, esophageal, renal and glioma tumor cells [12-15]. Thus, metformin has been recognized as not only an antidiabetic drug but a potentially preventive and therapeutic anticancer drug [9, 12, 13, 16].

MicroRNAs (miRNAs) are a class of small, noncoding, single-stranded RNAs that negatively regulate gene expression by mainly binding to 3′ untranslated region (UTR) of target mRNAs at the posttranscriptional level [17, 18]. Numerous researches have demonstrated that aberrant expression of miRNAs are involved in the development, invasion, metastasis and prognosis of various cancers [19-22]. According to recent studies [12, 23-25], the expression profiles of miRNAs are altered by metformin in different tumor cells, indicating that miRNAs might play an important role in the anticancer effect of metformin [25, 26].

In this study, we have reported that metformin had anticancer effect on cholangiocarcinoma through regulation of Drosha-mediated multiple carcinogenic miRNAs expression. And compositive assessment of these miRNAs expression changes could be more efficient to predict the prognosis for CCA patients and might be the novel anticancer therapeutic targets.

## RESULTS

### Metformin closely correlated with clinicopathologic characteristics and prognosis of CCA patients

**Figure 1:**
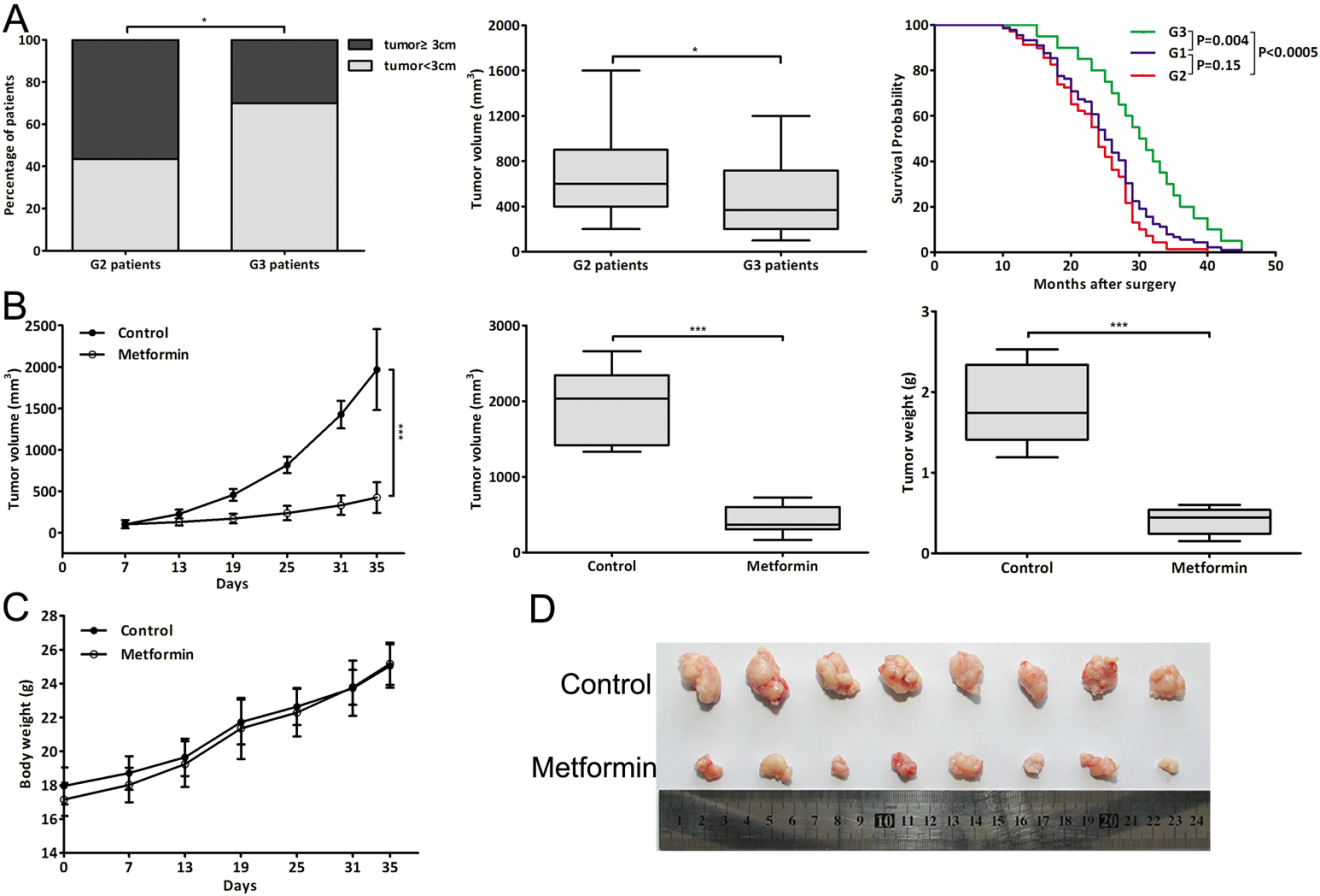
Metformin closely correlated with clinicopathologic characteristics and prognosis of CCA patients and inhibited CCA tumor growth *in vivo* (A) Percentage of patients with tumor size ≥3 cm or <3 cm; tumor volume of G2 and G3 patients; Kaplan-Meier plot of postoperative survival of three groups patients. (B) The tumor growth curve, tumor volume and weight of metformin-treated mice (2mg/mouse) and control mice (PBS). (C) The body weight of metformin-treated mice and control mice throughout the course of treatment. (D) Dissected tumor tissues. Data were expressed as mean±SD. *P<0.05, ***P<0.0005.

A total of 89 CCA patients were studied and the clinicopathologic characteristics of the patients were summarized in Supplementary Table 1. The tumor size and volume of G3 patients were smaller than those of G2 patients and the postoperative survival of G3 patients was longer than that of G2 or G1 patients (Fig. 1A). The representative HE staining of tumor tissues was shown in Fig. 10. The results showed that metformin treatment was closely associated with a decrease in the tumor size and volume of CCA patients, and the improved postoperative survival.

### Metformin inhibited CCA tumor growth *in vivo*

We evaluated the effect of metformin *in vivo* in a xenograft tumor model through metformin injection (2mg/mouse). Throughout the course of treatment, there were no visible side effects or changes in body weight of the mice (Fig. 1C). And metformin was significantly effective in inhibiting tumor growth *in vivo*, resulting in decreased tumor volume and weight (Fig. 1B, 1D). These data indicated that metformin could also reduce tumor volume and growth rate of CCA cells *in vivo*.

### Metformin inhibited CCA cells proliferation by cell cycle arrest

**Figure 2:**
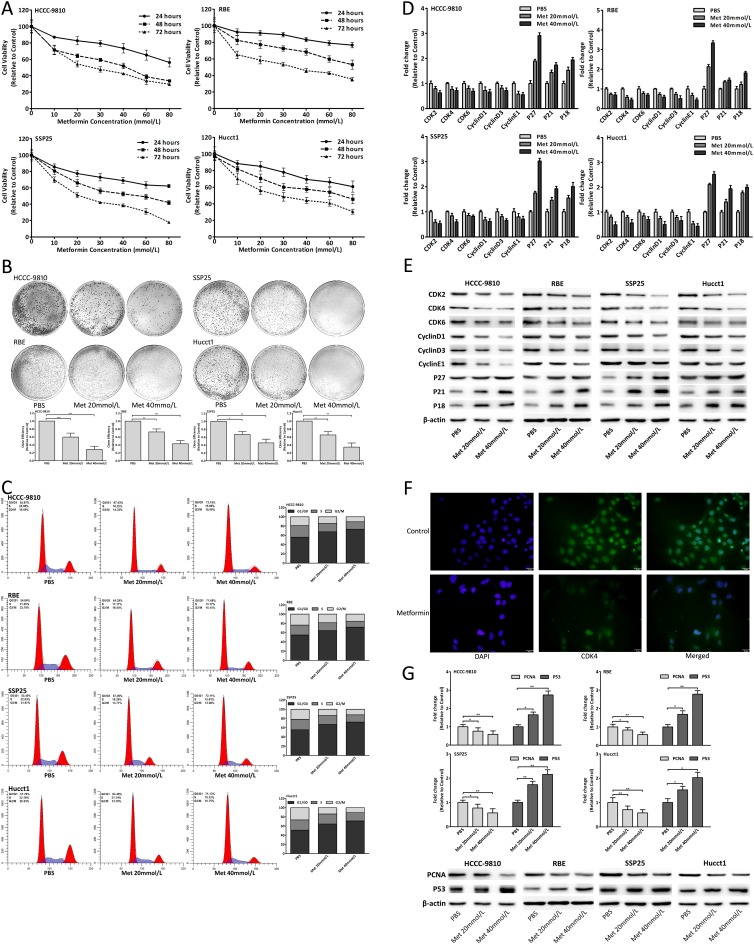
Metformin inhibited CCA cells proliferation by cell cycle arrest (A) Cell viability of CCA cells treated with metformin. (B) Colony formation of CCA cells after metformin treatment. (C) Cell cycle analysis of CCA cells treated with metformin for 48 hours. (D-E) mRNA and protein expression changes of cell-cycle-regulatory genes in CCA cells treated with metformin for 48 hours. (F) IF images showed CDK4 expression in RBE cells treated with 20mmol/L metformin for 48 hours. (G) mRNA and protein expression changes of PCNA and P53 in CCA cells after 48 hours metformin treatment. Data were expressed as mean±SD. The results were representative of three independent experiments. *P<0.05, **P<0.005, ***P<0.0005.

To investigate the effect of metformin on CCA *in vitro*, we used four human CCA cell lines including HCCC-9810, RBE, SSP25 and Hucct1. Metformin obviously decreased the viability of CCA cells in a dose-dependent manner (Fig. 2A) and 20, 40mmol/L metformin was selected for further study. Metformin also decreased colony formation of CCA cells in a dose-dependent manner (Fig. 2B). As shown in Fig. 2C, the cell cycle of CCA cells was arrested in G0/G1 phase by metformin in a dose-dependent manner. The mRNA and protein expression changes of cell-cycle-regulatory genes after metformin treatment were shown in Fig. 2D-E. IF assay showed that the cell count and CDK4 expression both decreased in metformin treated cells (Fig. 2F). Moreover, the proliferative marker genes PCNA and P53 were also detected after metformin treatment (Fig. 2G). The results illustrated that metformin blocked cell cycle to inhibit CCA cells proliferation.

### Metformin altered the miRNAs expression

**Figure 3:**
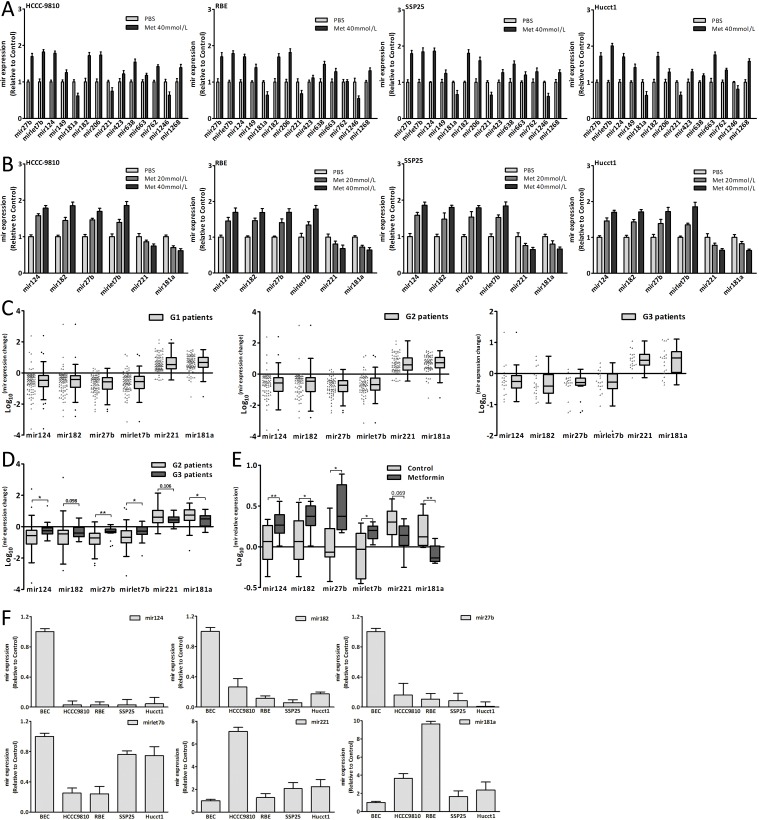
Metformin altered miRNAs expression (A) The expression changes of fourteen miRNAs in CCA cells treated with metformin for 48 hours. (B) The expression changes of mir124, 182, 27b, let7b, 221 and 181a in CCA cells treated with metformin for 48 hours. (C) The six miRNAs expression changes in three groups patients. (D) The six miRNAs expression changes of G2 patients compared with those of G3 patients. (E) The six miRNAs expression of metformin group and control group in xenograft tumor tissues. (F) The six miRNAs relative expression in CCA cells and BEC. Data were the mean±SD. *P<0.05, **P<0.005.

Recent studies reported that the miRNAs expression was altered by metformin [12, 23-26]. According to microassay platform analysis [12, 23, 24], the fourteen miRNAs which were obviously changed after metformin treatment were detected (Fig. 3A). The results showed that metformin also altered the miRNAs expression in CCA cells. Among these fourteen candidates, six miRNAs (hsa-mir-124-3p, hsa-mir-182-5p, hsa-mir-27b-3p, hsa-mir-let7b-5p, hsa-mir-221-3p and hsa-mir-181a-3p), which might target cell-cycle-regulatory genes according to bioinformatic algorithms, were selected for further study. The six miRNAs expression changes were in a dose-dependent manner in CCA cells after metformin treatment (Fig. 3B). There were obvious expression changes of six miRNAs in tumor tissues compared with adjacent normal tissues of CCA patients (Fig. 3C). Fig. 3D showed that the expression changes of mir124, 27b, let7b and 181a of G3 patients were significantly less than those of G2 patients. In the xenograft tumor tissues, there were obvious differences of mir124, 182, 27b, let7b and 181a expression between metformin group and control group (Fig. 3E). The six miRNAs expression of CCA cells and BEC (biliary epithelial cell) was shown in Fig. 3F. The results indicated that metformin could alter these six miRNAs expression *in vitro* and *in vivo*, and lessen these miRNAs expression changes in human tumor tissues. These six miRNAs might be involved in the CCA genesis and the anticancer effect of metformin.

### Dysregulation of six miRNAs in CCA genesis

**Figure 4:**
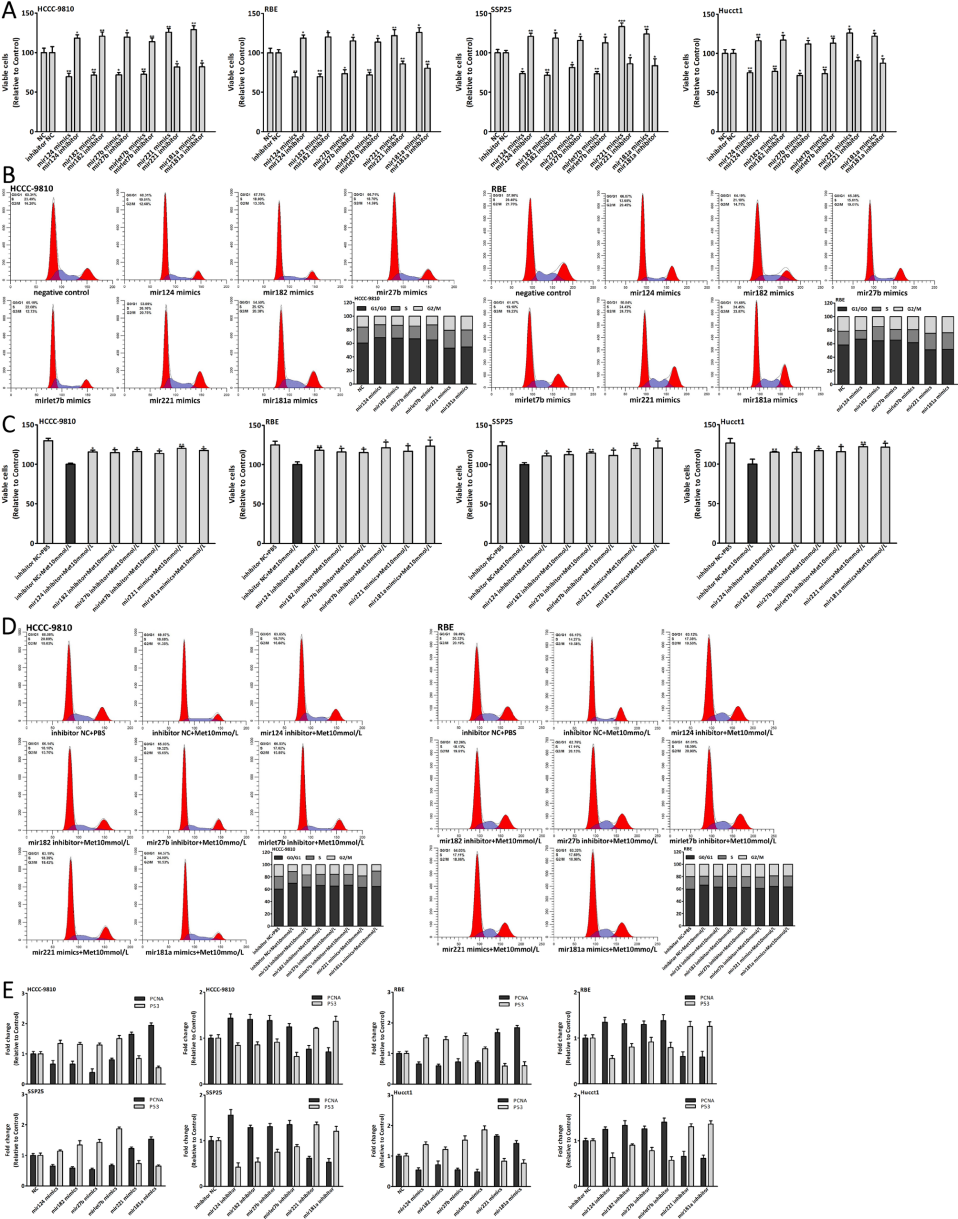
Dysregulation of six miRNAs in CCA genesis and regulating miRNAs could suppress the antiproliferative effect of metformin (A-B) Cell viability and cell cycle analysis of CCA cells 48 hours after miRNA mimics and inhibitor transfection. (C-D) Cell viability and cell cycle analysis of CCA cells which were transfected with miRNA mimics or inhibitor and treated with 10mmol/L metformin for 48 hours. (E) The expression changes of PCNA and P53 in CCA cells 48 hours after miRNA mimics and inhibitor transfection. Data were expressed as the mean±SD. *P<0.05, **P<0.005, ***P<0.0005.

The data showed that the six miRNAs were dysregulated in CCA (Fig. 3C, 3F). Thus we investigated the effect of these miRNAs on the CCA genesis through transfecting CCA cells with miRNA mimics and inhibitor. Upregulation of mir124, 182, 27b and let7b could inhibit the CCA cells proliferation and arrest the cell cycle in G0/G1 phase, however, the proliferation and cell cycle were both promoted through upregulating mir221 or mir181a expression (Fig. 4A-B). PNCA and P53 were also detected after transfection (Fig. 4E). The results further confirmed that these dysregulated miRNAs, including downregulated mir124, 182, 27b, let7b and upregulated mir221, 181a, were involved in the CCA genesis and promoted this process.

### Regulating miRNAs could suppress the antiproliferative effect of metformin

Metformin altered the expression of six miRNAs which could inhibit the CCA cells proliferation, indicating that the antiproliferative effect of metformin might be suppressed by regulating miRNAs expression. Consequently, we treated the CCA cells, which had already been transfected with miRNA mimics or inhibitor, with 10mmol/L metformin. The data of cell viability assay and cell cycle analysis showed that the antiproliferation and cell cycle arrest induced by metformin were reversed (Fig. 4C-D). The results further confirmed that these miRNAs were involved in the anticancer effect of metformin.

### The six miRNAs targeted multiple cell-cycle-regulatory genes

**Figure 5:**
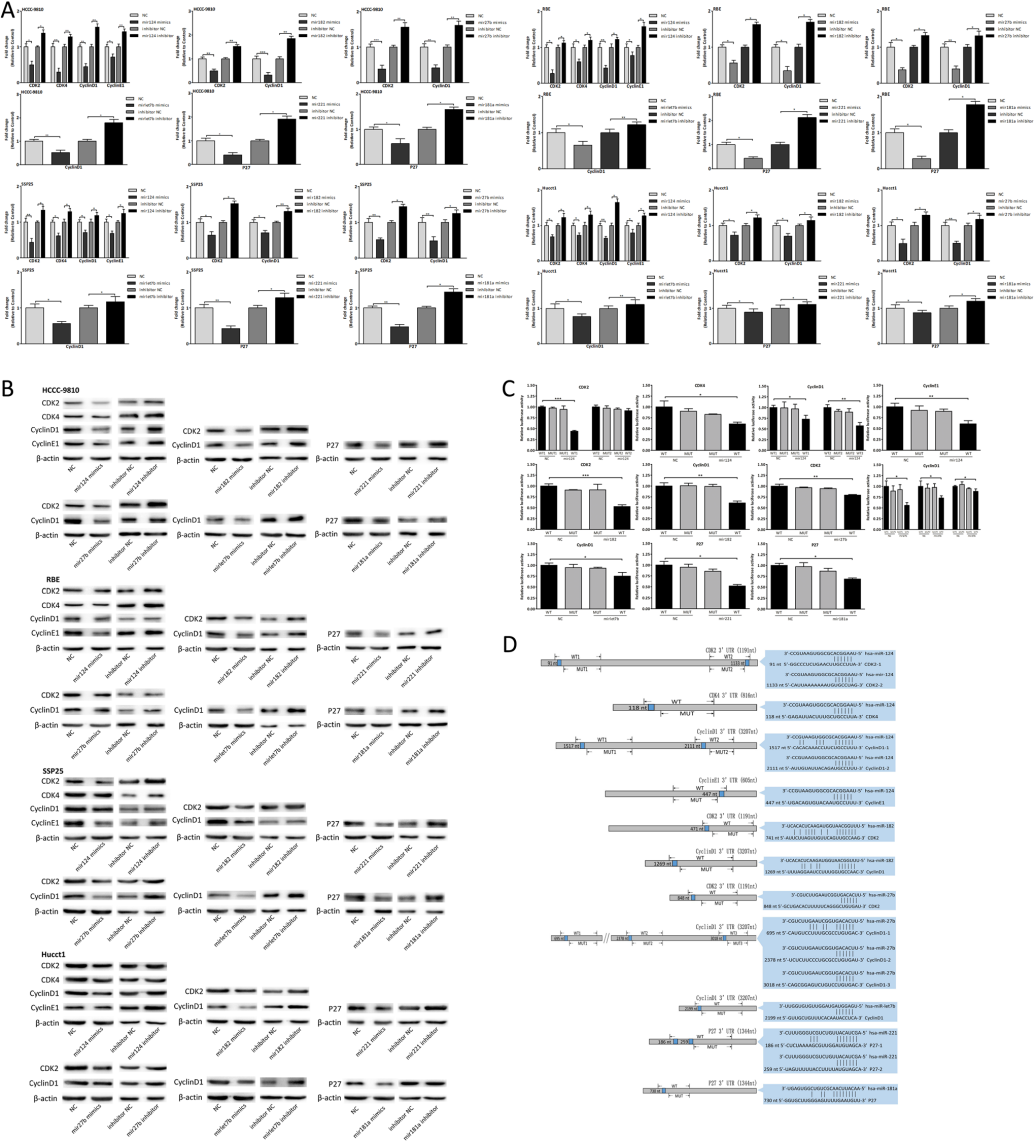
The six miRNAs targeted multiple cell-cycle-regulatory genes (A-B) mRNA and protein expression changes of putative target genes in CCA cells 48 hours after miRNA mimics and inhibitor transfection. (C) Luciferase activity of 293T cells co-transfected with miRNA mimics and indicated WT or MUT reporter vector. (D) Schematic presentation of the sequence sites of miRNAs and binding UTRs, and pattern of WT and MUT reporter vectors. Data were expressed as mean±SD. The results were representative of three independent experiments. *P<0.05, **P<0.005, ***P<0.0005.

Next we assessed whether these miRNAs could target the putative cell-cycle-regulatory genes. We transfected the CCA cells with miRNA mimics and inhibitor to detect the mRNA and protein expression changes of putative target genes (Fig. 5A-B). The results of luciferase activity assay were shown in Fig. 5C. The sequence sites of miRNA and binding UTR of target genes and structure of reporter vectors were shown in Fig. 5D. In summary, the cell-cycle-regulatory genes CDK2, CDK4, CyclinD1 and CyclinE1 were targeted by mir124, CDK2 and CyclinD1 were targeted by mir182 and mir27b, CyclinD1 was targeted by mirlet7b, P27 was targeted by mir221 and mir181a.

### The miRNAs expression correlated with prognosis of CCA patients

**Figure 6:**
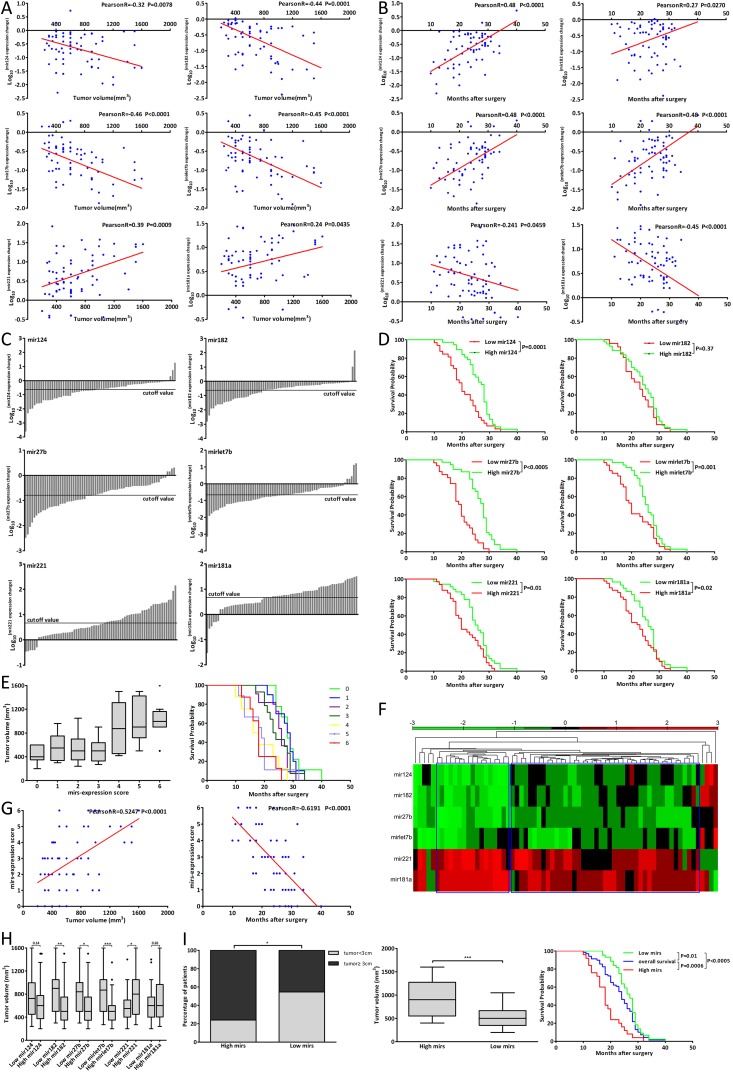
The miRNAs expression correlated with prognosis of CCA patients (A-B) The correlation analysis between tumor volume, postoperative survival and each miRNA expression changes of G2 patients. (C) The cutoff value of each miRNA expression changes of G2 patients. (D) The postoperative survival of Low and High mir patients of each miRNA. (E) The tumor volume and postoperative survival of different mirs-expression score patients. (F) Hierarchical clustering analysis of G2 patients according to six miRNAs expression changes. (G) The correlation analysis between mir-expression score and tumor volume, postoperative survival of G2 patients. (H) The tumor volume of Low mir patients compared with that of High mir patients. (I) The tumor size, tumor volume and postoperative survival of Low mirs patients compared with those of High mirs patients. Data were expressed as mean±SD. *P<0.05, **P<0.005, ***P<0.0005.

The six miRNAs were found to be involved in the CCA genesis and the anticancer effect of metformin, thus we wanted to evaluate whether these miRNAs were clinically useful for CCA patients. The expression change of mir182, 27b and let7b was closely associated with tumor volume of G2 patients (Fig. 6A, R<-0.4). Fig. 6B showed that the expression change of mir124, 27b, let7b and 181a was also closely associated with postoperative survival of G2 patients (R≤-0.45 or ≥0.45).

The mean Log_10_^(mir expression change)^ was defined as the cutoff value of each miRNA expression change [27-30] (Fig. 6C). According to each miRNA expression change which was greater or less than its cutoff value, the patient was classified into High or Low mir patient [31-34]. And G2 patients could be divided into two subgroups, such as Low mir124 patients and High mir124 patients. Fig. 6H showed that there were significant differences in tumor volume between Low and High mir patients of mir182, 27b, let7b and 221. The postoperative survival of Low and High mir patients was listed in Supplementary Table 3 and there were significant differences of postoperative survival between Low and High mir patients of mir124, 27b, let7, 221 and 181a (Fig. 6D).

Further we applied mirs-expression score to combine six miRNAs expression changes together. In one patient, the expression change of mir124, 182, 27b or let7b was less than its cutoff value and this single miRNA expression of the patient scored one; the expression change of mir221 or 181a was greater than its cutoff value and this single miRNA expression of the patient also scored one. According to six miRNAs expression changes, the mirs-expression score of one single patient might range from 0 to 6. Fig. 6E showed the tumor volume and postoperative survival of different score patients and mirs-expression score was closely associated with the tumor volume and postoperative survival of G2 patients (Fig. 6G).

The patient with mirs-expression score from 0 to 3 was considered as Low mirs patient and the patient with mirs-expression score from 4 to 6 was considered as High mirs patient. Accordingly, G2 patients were divided into two subgroups including Low mirs patients (n=44) and High mirs patients (n=25). Based on these six miRNAs expression changes, we applied hierarchical clustering analysis to cluster G2 patients. As shown in Fig. 6F, G2 patients were also clustered into two subgroups which were mostly consistent with the results by using mirs-expression score. The detailed clinicopathologic characteristics of Low and High mirs patients were listed in Supplementary Table 2. Fig. 6I showed that the tumor size and volume of Low mirs patients were less than those of High mirs patients and there were the most significant differences of tumor volume and postoperative survival between Low and High mirs patients. These data showed that the associations with tumor volume and postoperative survival were stronger for mirs-expression than for single miRNA expression, and the differences of tumor volume and postoperative survival between Low and High mirs patients were more significant than those between single Low and High mir patients. These results suggested that these miRNAs expression could be used to predict the prognosis for CCA patients and assessment of multiple miRNAs expression might be more efficient.

### Metformin modulated Drosha to affect miRNAs expression

**Figure 7:**
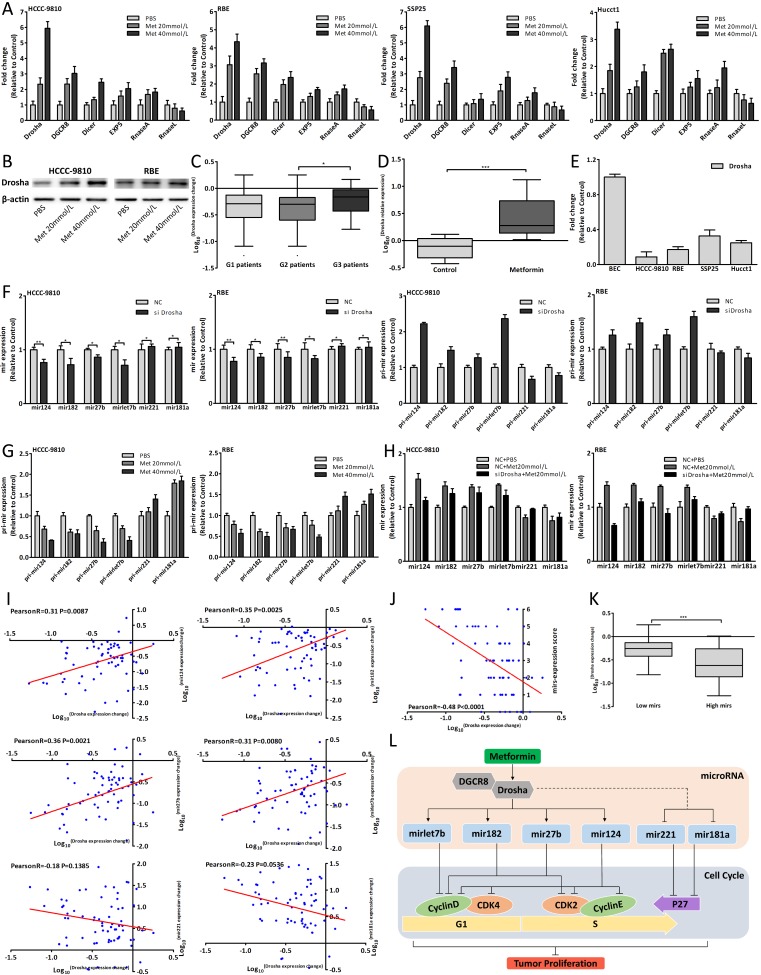
Metformin modulated Drosha to affect miRNAs expression (A) Drosha, DGCR8, Dicer, EXP5, RanseA and RnaseL expression changes in CCA cells after 48 hours metformin treatment. (B) Drosha protein expression changes in CCA cells treated with metformin for 48 hours. (C) Drosha expression change in three groups patients. (D) Drosha expression of metformin group and control group in xenograft tumor tissues. (E) Drosha relative expression in CCA cells and BEC. (F) The mature and pri-miRNAs expression changes in CCA cells 48 hours after Drosha siRNA transfection. (G) The pri-miRNAs expression changes in CCA cells after 48 hours metformin treatment. (H) The miRNAs expression changes of CCA cells transfected with Drosha siRNA and treated with 20mmol/L metformin for 48 hours. (I) The correlation analysis between Drosha expression change and each miRNA expression change in G2 patients. (J) The correlation analysis between Drosha expression change and mir-expression score. (K) Drosha expression of Low mirs patients compared with that of High mirs patients. (L) Schematic model of the underlying anticancer mechanism of metformin in CCA. Data were expressed as mean±SD. The results were representative of three independent experiments. *P<0.05, **P<0.005, ***P<0.0005.

**Figure 8:**
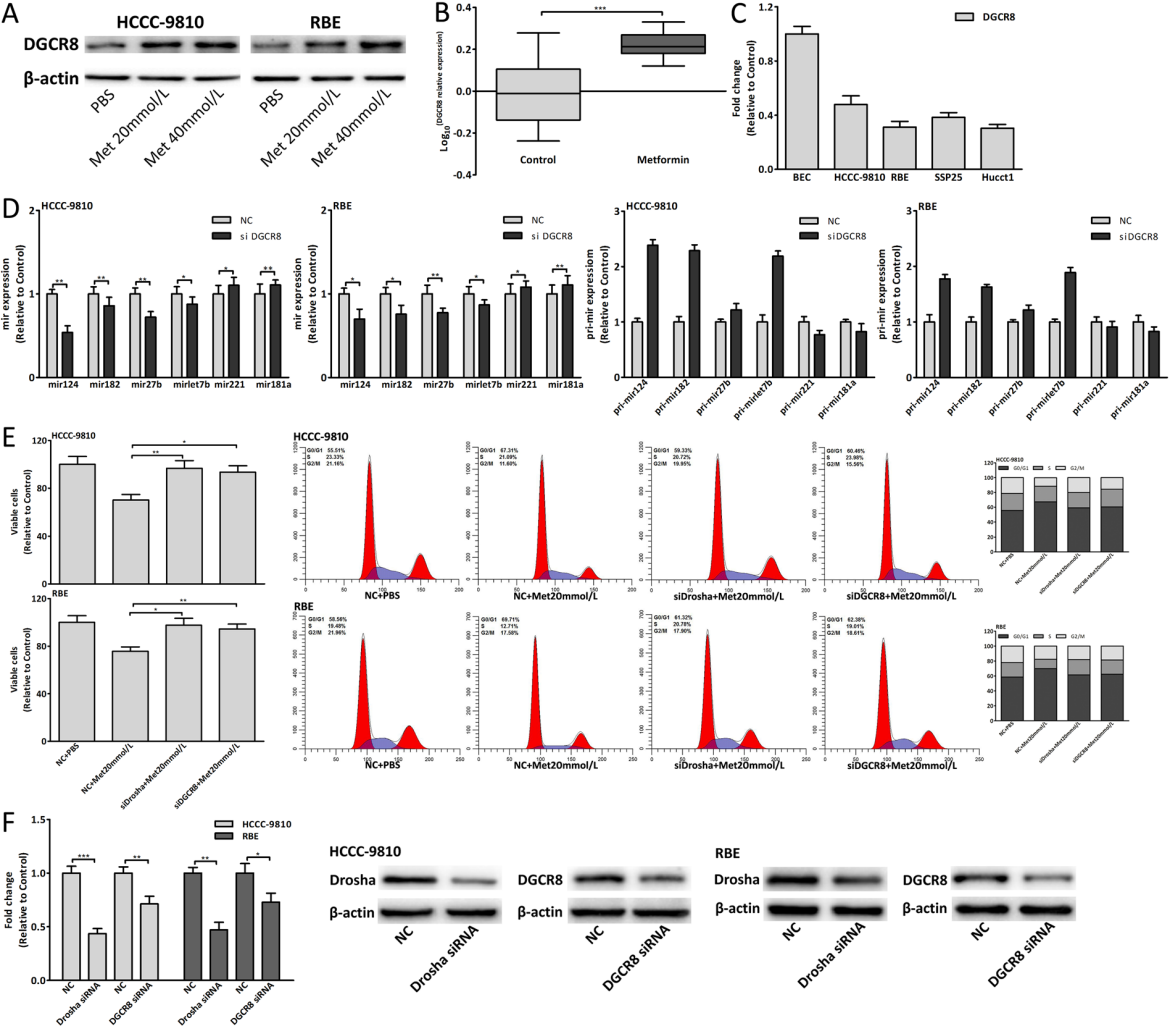
Metformin also upregulated DGCR8 to affect miRNAs expression (A) DGCR8 protein expression change in CCA cells treated with metformin for 48 hours. (B) DGCR8 expression of metformin group and control group in xenograft tumor tissues. (C) DGCR8 relative expression in CCA cells and BEC. (D) The mature and pri-miRNAs expression changes in CCA cells 48 hours after DGCR8 siRNA transfection. (E) Cell viability and cell cycle analysis of CCA cells transfected with Drosha/DGCR8 siRNA and treated with 20mmol/L metformin for 48 hours. (F) The siRNA efficiency of Drosha and DGCR8. Data were expressed as mean±SD. The results were representative of three independent experiments. *P<0.05, **P<0.005, ***P<0.0005.

To understand how metformin altered the miRNAs expression, we detected the key enzymes expression of miRNA biogenesis and Drosha was most obviously upregulated [35-38] (Fig. 7A-B). Metformin also upregulated Drosha expression in human and xenograft tumor tissues (Fig. 7C-D). Drosha expression of CCA cells and BEC was shown in Fig. 7E. Drosha could promote the process of pri-miRNA to mature-miRNA and Fig. 7F showed the expression changes of mature and pri-miRNAs after Drosha siRNA transfection by using qRT-PCR [35, 39, 40]. The six pri-miRNAs expression changes, which were opposite to expression changes of mature-miRNAs, were in a dose-dependent manner after metformin treatment (Fig. 7G, 3B). Further we found that downregulated Drosha could lessen the miRNAs expression changes induced by metformin (Fig. 7H) and suppress antiproliferative and cell cycle arrest effect of 20mmol/L metformin (Fig. 8E). In human tissues, Drosha expression change was positively correlated with the expression changes of mir124, 182, 27b and let7b (Fig. 7I), and negatively associated with mirs-expression score (Fig. 7J). Drosha expression of Low mirs patients was higher than that of High mirs patients (Fig. 7K). In brief, these results indicated that metformin upregulated Drosha, which could modulate the miRNA biogenesis, to affect these miRNAs expression. The schematic presentation of the underlying anticancer mechanism of metformin was shown in Fig. 7L.

### Metformin also upregulated DGCR8 to affect miRNAs expression

Drosha usually combined DGCR8 to form a complex to function, thus DGCR8 was also studied. Metformin upregulated DGCR8 expression both *in vitro* and *in vivo* (Fig. 7A, 8A-B). Downregulated DGCR8 interfered by siRNA could affect the mature and pri-miRNAs expression and the expression changes of mature-miRNAs were opposite to those of pri-miRNAs (Fig. 8D). As with Drosha, downregulated DGCR8 could also suppress antiproliferative and cell cycle arrest effect of 20mmol/L metformin (Fig. 8E). These results were consistent with the research in Drosha and implied that metformin also upregulated DGCR8 to modulate these miRNAs expression. The siRNA efficiency of Drosha and DGCR8 was shown in Fig. 8F.

### Dysregulation of Drosha/DGCR8 in CCA genesis

**Figure 9:**
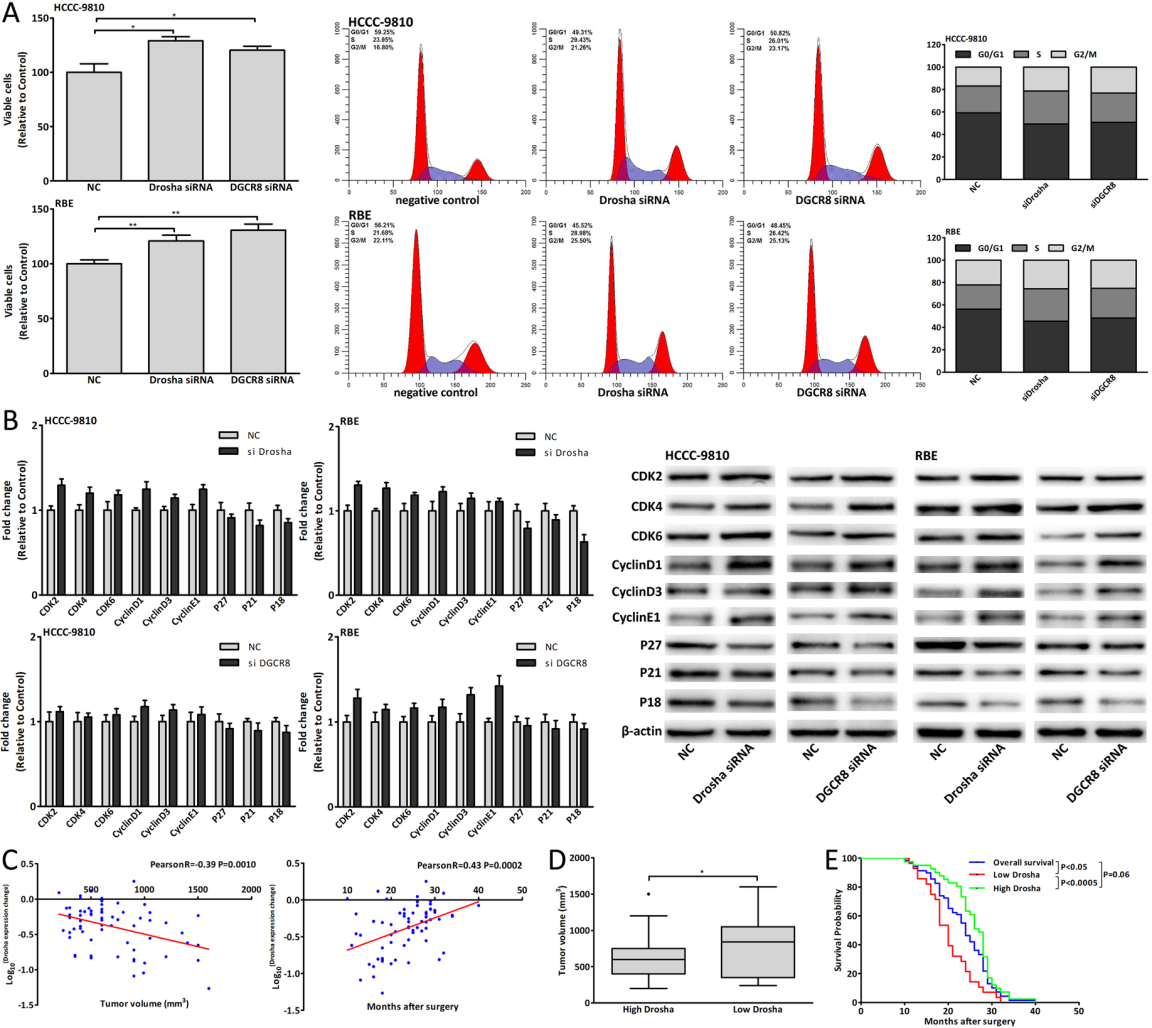
Dysregulation of Drosha/DGCR8 in CCA genesis (A) Cell viability and cell cycle analysis of CCA cells 48 hours after Drosha/DGCR8 siRNA transfection. (B) mRNA and protein expression changes of cell-cycle-regulatory genes in CCA cells 48 hours after Drosha/DGCR8 siRNA transfection. (C) The correlation analysis between Drosha expression change and tumor volume, postoperative survival of G2 patients. (D-E) The tumor volume and postoperative survival of High Drosha patients compared with those of Low Drosha patients. Data were expressed as mean±SD. The results were representative of three independent experiments. *P<0.05, **P<0.005.

We found that Drosha was downregulated in the tumor tissues compared with adjacent normal tissues of CCA patients (Fig. 7C). Drosha and DGCR8 expression in CCA cells was relatively less than those in BEC (Fig. 7E, 8C). Using siRNA to knock down Drosha/DGCR8 *in vitro*, the proliferation and cell cycle of CCA cells were both promoted (Fig. 9A). Fig. 9B showed the mRNA and protein expression changes of cell-cycle-regulatory genes after Drosha/DGCR8 siRNA transfection. Drosha expression change was negatively associated with tumor volume and positively correlated with postoperative survival of G2 patients (Fig. 9C). As with miRNAs, the mean Log_10_^(Drosha expression change)^ was defined as the cutoff value of Drosha expression change of G2 patients and High Drosha patients had smaller tumor volume and longer postoperative survival (Fig. 9D-E). These results indicated that dysregulated Drosha/DGCR8, which upstream regulated these carcinogenic miRNAs expression, promoted the CCA genesis and Drosha expression might be also used to predict the prognosis for CCA patients.

**Figure 10:**
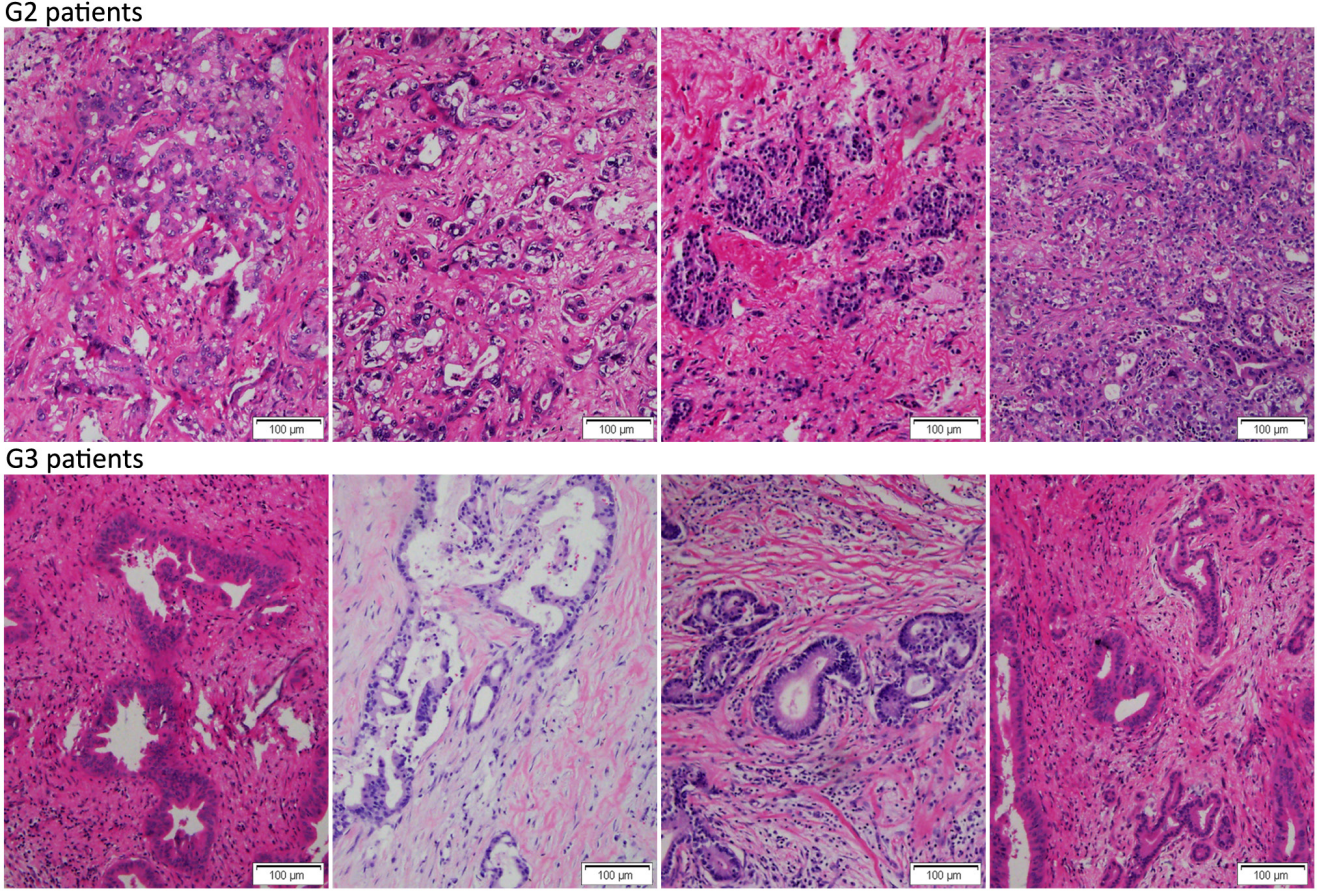
All collected CCA samples were stained with HE Representative images showed the tumor tissues HE staining of G2 and G3 patients.

## DISCUSSION

Previous studies have pointed out that metformin was associated with reduced risk and improved prognosis of many cancers and had an excellent safety record in diabetic patients; metformin also inhibited various tumor cells proliferation both *in vitro* and *in vivo* [9-16, 23, 24, 26]. Recent studies showed that metformin altered the expression profiles of miRNAs in several tumor cells [12, 23, 24]. These findings suggested that metformin had potent anticancer effect which miRNAs might be involved in and might be a promising strategy for cancer prevention and treatment. In this study, we showed the anticancer effect of metformin in cholangiocarcinoma and studied the underlying anticancer mechanism of metformin. We found that metformin treatment was closely associated with clinicopathological characteristics of CCA patients and the improved postoperative survival. Using *in vitro* and *in vivo* assays, we confirmed that metformin arrested cell cycle in G0/G1 phase to inhibit CCA cells proliferation.

To investigate whether miRNAs were involved in the anticancer effect of metformin, we identified six miRNAs which were altered by metformin *in vitro* and *in vivo*, and the targets of these miRNAs were all cell-cycle-regulatory genes. It was found that there were obvious differences of these miRNAs expression in tumor tissues compared with adjacent normal tissues and the dysregulation of these miRNAs could promote the cell cycle and enhance the CCA cells proliferation. According to researches [20, 21], these miRNAs and their targets were also dysregulated and promoted the carcinogenesis in other cancers [34, 41]. The miRNAs expression changes induced by metformin were opposite to the expression changes of these dysregulated miRNAs in human tumor tissues and metformin could lessen dysregulated expression changes of these miRNAs. And regulating miRNAs expression could suppress the antiproliferative and cell cycle arrest effect of metformin. These results implicated that these dysregulated miRNAs were involved in the CCA genesis and promoted this process. Metformin exactly modulated these carcinogenic miRNAs, which further targeted cell-cycle-regulatory genes to arrest the cell cycle and inhibit the proliferation, to achieve the anticancer effect in cholangiocarcinoma.

Another finding of this study was that these miRNAs expression changes were clinically useful and could be used to predict the prognosis for CCA patients. We found that there were various degrees of correlations between tumor volume, postoperative survival and each miRNA expression change. Based on the cutoff value of each miRNA expression change [27-29, 31], G2 patients were divided into High and Low mir patients, and High mir patients of mir124, 182, 27b, let7b and Low mir patients of mir221, 181a had smaller tumor volume and longer postoperative survival. Meanwhile, we utilized mirs-expression score to compositely evaluate six miRNAs expression in one single patient and classified G2 patients into two subgroups including High and Low mirs patients, which were mostly consistent with the results of hierarchical clustering analysis. The results proved that the associations with tumor volume and postoperative survival were stronger for composite six miRNAs assessment than for single miRNA evaluation; and the differences of tumor volume and postoperative survival between High and Low mirs patients were more significant than those between single High and Low mir patients. In addition, Low mirs patients had the smallest tumor volume and the longest postoperative survival. These data illustrated that to evaluate multiple miRNAs expression might be more efficacious for assessing the prognosis of CCA patients.

To understand how metformin affected the miRNAs expression, we detected the key enzymes expression of miRNA biogenesis in metformin treated CCA cells and Drosha had the most obviously increased expression [35-38]. Metformin also increased Drosha expression in human and xenograft tumor tissues. The increased Drosha expression induced by metformin was similar to the major of six miRNAs expression changes after metformin treatment *in vitro* and *in vivo*. The miRNAs expression changes induced by regulating Drosha were consistent with the miRNAs expression changes after metformin treatment, and downregulating Drosha could lessen the miRNAs expression changes induced by metformin. Compared with regulating single miRNA, downregulated Drosha could suppress antiproliferative and cell cycle arrest effect of relative high concentration metformin. We also verified the pri-miRNAs expression changes after metformin treatment and Drosha siRNA transfection and analyzed the correlations between Drosha expression and miRNAs expression in human tissues. However, it was found that Drosha did not modulate these miRNAs expression directly and the expression changes of mir221 and 181a might be due to unbalanced miRNA biogenesis which was affected by increased Drosha [42]. Drosha usually combined with DGCR8 to function and thus DGCR8 was also studied [35, 38, 43]. Taken together, the results indicated that metformin affected these miRNAs expression through upregulating Drosha which upstream modulated the miRNAs biogenesis directly or indirectly.

In summary, our data presented in this study provided evidence elucidating that metformin, a widely used antidiabetic drug, could inhibit CCA tumor growth and improve the prognosis of CCA patients. The underlying anticancer mechanism of metformin depended on the regulation of Drosha-mediated multiple carcinogenic miRNAs which could directly target cell-cycle-regulatory genes to arrest the cell cycle and inhibit the proliferation. We also discovered that compositive assessment of multiple miRNAs expression could be more efficient to predict the prognosis for CCA patients and might be the potential therapeutic targets for CCA prevention and treatment. Further work should be performed to get more understanding about anticancer mechanisms of metformin, and to explore the clinical use of metformin for cancer therapy.

## MATERIALS AND METHODS

### Patients and tissue samples

A total of 89 CCA specimens were obtained from the Second Affiliated Hospital of Harbin Medical University from 2006 to 2011. All the patients were diagnosed with extrahepatic cholangiocarcinoma and confirmed pathologically, and received the tumor resection. None of the patients had received chemotherapy or radiotherapy preoperatively or postoperatively. All the patients received the same medical care and follow up. Samples were collected according to the approval of the ethical committee of the Second Affiliated Hospital of Harbin Medical University, with written individual consent from each patient for this study. The total 89 CCA patients were defined as Group One (G1) patients. Among these 89 patients, 69 patients without diabetes and metformin treatment were defined as Group Two (G2) patients and 20 patients who received metformin treatment before the surgery because of type II diabetes were defined as Group Three (G3) patients. The detailed clinicopathologic characteristics of the CCA patients in this study were listed in Supplementary Table 1.

### Reagents and antibodies

Metformin was purchased from Sigma-Aldrich. DMEM, RPMI-1640, Lipofectamine 2000, TRIzol, penicillin/streptomycin, trypsin and fetal bovine serum (FBS) were from Invitrogen. MicroRNAs mimics and inhibitor, and siRNA against Drosha and DGCR8 were purchased from GenePharma (Shanghai, China). The antibodies used in western blot were listed in Supplementary Table 4.

### Cell lines and culture

HCCC-9810, RBE and SSP25 were purchased from the Type Culture Collection of the Chinese Academy of Sciences (Shanghai, China); Hucct1, 293T and human biliary epithelial cell (BEC) were preserved in our laboratory. Cells were grown in DMEM or RPMI-1640 supplemented with 10% FBS and penicillin/streptomycin (100 U/mL/50mg/mL) at 37°C and 5% CO_2_. The cells had been passed for less than six months in culture when the experiments were carried out.

### Target genes prediction of miRNAs

The microRNA target bioinformatics software microRNA.org was used to predict the target genes of miRNAs. The miRNAs and putative targeted cell-cycle-regulatory genes were listed in Supplementary Table 5.

### RNA extraction and qRT-PCR

Total RNA was extracted by using TRIzol reagent. Reverse transcription reactions were carried out using M-MLV reverse transcriptase (Invitrogen) and qRT-PCR (quantificational real-time PCR) was performed on Applied Biosystem as described previously [44, 45]. The relative expression levels of miRNAs and mRNAs were calculated and quantified using the 2^−ΔΔCt^ method after normalization for the expression of control, human U6 snRNA and β-actin served as endogenous controls. The primers used in qRT-PCR were listed in Supplementary Table 6.

### Cell viability, colony formation assay and western blot

Cell viability and colony formation assay were carried out as previously described [46]. Total protein was extracted using RIPA buffer and protein expression was analyzed by western blot as described previously [46]. β-actin served as an endogenous control.

### Flow cytometric analysis

Cells were harvested, fixed in 80% ethanol and stored at 4°C. On the day of analysis, cells were washed and centrifuged using cold PBS, suspended in 10ml PBS and 1ml RnaseA solution (250mg/ml) followed by incubation for 30 minutes at 37°C. Then, 100μl propidium iodide stain (100mg/ml) was added to each tube, which was then incubated at 4°C for 30 minutes prior to analysis. Flow cytometric analysis was conducted using BD LSRFortessa (BD Biosciences). The percentages of cells in different phases of cell cycle were analyzed by using FlowJo software.

### Immunofluorescence (IF) analysis

Cells were rinsed with PBS and fixed with 4% paraformaldehyde for 30 minutes at room temperature followed by permeabilisation with 0.1% sodium citrate plus 0.1% Triton X-100. The cells were subjected to immunofluorescent staining with CDK4 antibody (1:200, CST #12790) for 16 hours at 4°C. Cells were then washed with cold PBS three times for five minutes each and incubated with fluorescence labeled secondary antibody (1:400, #ZF0511, ZSGB-BIO) for 30 minutes. The cells were visualized using inverted fluorescence microscope (FSX100, Olympus).

### Luciferase activity assay

The putative binding site of target genes 3′-UTR sequences, the downstream of which contained the firefly luciferase gene, were cloned and inserted into the SpeI and HindIII site of pMIR-REPORT luciferase vector (Ambion). The pMIR-target genes-3′ UTR mutants that deleted the putative binding site were also generated. The primers for luciferase report vectors were listed in Supplementary Table 7. The WT or MUT pMIR-REPORT luciferase vector was transfected into 293T cells with miRNA mimics and pRL-TK vector (Promega) was used for normalization of transfection efficiency. The firefly luciferase activity was measured as described previously [46].

### Subcutaneous CCA experiments

Female nude mice (5-6 weeks old) were purchased from Beijing Vital River Laboratory Animal, Inc. (Beijing, China). All animal experiments were done according to the Institutional Animal Care and Use Committee-approved protocol. Institutional guidelines for the proper and humane use of animals in research were followed. The mice were maintained in specific pathogen-free environment. 5×10^6^ Hucct1 cells suspended in PBS were inoculated subcutaneously in the flanks. Metformin treatment was started 7 days after inoculation of the cells. The experimental group (n=8) was treated daily with i.p. injection of metformin (2mg/mouse, administered in PBS) for four weeks, whereas the control group (n=8) received equal volume of PBS only. Throughout the course of treatment, the mice were monitored daily and weighed every third day to check for physical condition. The tumor volume was calculated using the formula: length×(width)^2^×0.5.

### Statistical analysis

Statistical analysis was performed with the SPSS 16.0 software. Values were expressed as mean±SD using the Student's *t*-test. The postoperative survival was compared with the Kaplan-Meier method and the significance was determined by the log-rank test. The correlation analysis was examined using spearman rank correlation test. The *P*<0.05 was considered to be statistically significant.

## SUPPLEMENTARY MATERIAL TABLES


