# Supplementary material for: Metformin inhibits tumor growth by regulating multiple miRNAs in human cholangiocarcinoma
Source: Oncotarget. 2014 Dec 18;6(5):3178–94. doi: 10.18632/oncotarget.3063 (PMC4413646; doi:10.18632/oncotarget.3063)
Supplement: Supplementary file 1 [file oncotarget-06-3178-s001.pdf]

Metformin inhibits tumor growth by regulating multiple miRNAs in human cholangiocarcinoma

Supplementary Material

Supplementary Table 1: Clinicopathologic characteristics of 89 CCA patients

Parameter	Group One (n=89)	Group Two (n=69)	Group Three (n=20)	P value
Gender [n (%)]				
Male	47(52.81)	38(55.07)	9(45.00)	0.433
Female	42(47.19)	31(44.93)	11(55.00)	
Age [years; n (%)]				
<60	38(42.70)	31(44.93)	7(35.00)	0.231
≥60	51(57.30)	38(55.07)	13(65.00)	
Median age [years; mean (±SD)]	61.10(±10.33)	60.39(±10.57)	63.55(±9.63)	0.231
Weight [Kg; mean (±SD)]	63.24(±11.96)	62.09(±11.08)	67.18(±11.67)	0.094
Tobacco use [n (%)]	28(40.58)	24(34.78)	4(20.00)	0.214
Ethanol use [n (%)]	17(19.10)	13(18.84)	4(20.00)	0.909
HBV infection [n (%)]	9(10.11)	7(10.15)	2(10.00)	0.985
Lab finding [n (%)]				
Elevated CA19-9[a]	70(78.65)	55(79.71)	15(75.00)	0.655
Elevated CA125[b]	13(14.61)	11(15.94)	2(10.00)	0.513
Elevated CEA[c]	31(34.83)	23(33.33)	8(40.00)	0.587
Elevated AFP[d]	14(15.73)	10(14.49)	4(20.00)	0.557
Operative time [hours; mean (±SD)]	3.46(±1.54)	3.64(±1.64)	2.85(±1.21)	0.044
Blood loss [ml; mean (±SD)]	338(±301)	347(±330)	306(±228)	0.597
R0 resection[e] [n (%)]	67(75.28)	51(73.91)	16(80.00)	0.584
Margin status [n (%)]				
Positive	12(13.48)	10(14.49)	2(10.00)	0.609
Negative	77(86.52)	59(85.51)	18(90.00)	
Differentiations [n (%)]				
Well	23(25.84)	17(24.64)	6(20.00)	0.143
Moderate	42(47.19)	30(43.48)	12(60.00)	
Poor	24(26.97)	22(31.88)	2(10.00)	
Histological type [n (%)]				
Adenocarcinoma	80(89.89)	63(91.30)	17(85.00)	0.446
Mucinous adenocarcinoma	6(6.74)	4(5.80)	2(10.00)	
Papillary carcinoma	3(3.37)	2(2.90)	1(5.00)	
Lymph node invasion [n (%)]				
Present	31(34.83)	28(40.58)	3(15.00)	0.035
Absent	58(65.17)	41(59.42)	17(85.00)	
Organ invasion[f] [n (%)]				
Present	17(19.10)	15(21.74)	2(10.00)	0.109
Absent	72(80.90)	54(78.26)	18(90.00)	
Tumor size[g] [cm; n (%)]				
<3	44(49.44)	30(43.48)	14(70.00)	0.037
≥3	45(50.56)	39(56.52)	6(30.00)	
Tumor volume[h] [mm^3; mean (±SD)]	645(±365)	689(±349)	492(±383)	0.032
Hospital stay [days; mean (±SD)]	19.97(±10.79)	20.16(±11.54)	19.3(±9.93)	0.756
Survival [months; mean (±SD)]	25.03(±7.21)	23.48(±6.26)	30.4(±7.86)	<0.0005

Note: a. carbohydrate antigen 19-9, the reference range is less than 37 U/ml; b. carbohydrate antigen 125, the reference range is less than 35 U/ml; c. carcinoembryonic antigen, the reference range is less than 5 ng/ml; d. alpha-fetoprotein, the reference range is less than 13.4 ng/ml; e. complete resection with no microscopic residual tumor (margins are microscopically negative according to the pathologist); f. organs include liver, pancreas, duodenum, colon, omentum, portal vein and hepatic artery; g. the maximum diameter of tumor tissue; h. tumor volume= length×$(width)^2$×0.5

Supplementary Table 2: Clinicopathologic characteristics of Low and High mirs patients

Parameter	Low mirs patients (n=44)	High mirs patients (n=25)	P value
Gender [n (%)]			
Male	23(52.27)	15(60.00)	0.542
Female	21(47.73)	10(40.00)	
Age [years; n (%)]			
<60	23(52.27)	8(32.00)	0.107
≥60	21(47.73)	17(68.00)	
Median age [years; mean (±SD)]	59.36(±11.74)	62.59(±6.95)	0.237
Weight [Kg; mean (±SD)]	61.21(±11.07)	63.98(±11.44)	0.342
Tobacco use [n (%)]	15(34.09)	9(36.00)	0.875
Ethanol use [n (%)]	8(18.18)	5(20.00)	0.855
HBV infection [n (%)]	4(9.09)	3(12.00)	0.706
Lab finding [n (%)]			
Elevated CA19-9	34(77.27)	21(84.00)	0.511
Elevated CA125	6(13.64)	5(20.00)	0.495
Elevated CEA	15(34.09)	8(32.00)	0.862
Elevated AFP	6(13.64)	4(16.00)	0.792
Operative time [hours; mean (±SD)]	3.68(±1.54)	3.55(±1.75)	0.745
Blood loss [ml; mean (±SD)]	317(±238)	361(±356)	0.602
R0 resection [n (%)]	34(77.27)	17(68.00)	0.407
Margin status [n (%)]			
Positive	5(11.36)	5(20.00)	0.335
Negative	39(88.64)	20(80.00)	
Differentiations [n (%)]			
Well	13(29.55)	4(16.00)	0.293
Moderate	18(40.90)	12(48.00)	
Poor	13(29.55)	9(36.00)	
Histological type [n (%)]			
Adenocarcinoma	42(95.46)	21 (84.00)	0.283
Mucinous adenocarcinoma	1(2.27)	3(12.00)	
Papillary carcinoma	1(2.27)	1(4.00)	
Lymph node invasion [n (%)]			
Present	13(29.55)	15(60.00)	0.013
Absent	31(70.45)	10(40.00)	
Organ invasion [n (%)]			
Present	8(18.18)	7(28.00)	0.349
Absent	36(81.82)	18(72.00)	
Tumor size [cm; n (%)]			
<3	24(54.55)	6(24.00)	0.013
≥3	20(45.45)	19(76.00)	
Tumor volume [mm^3; mean (±SD)]	542(±220)	950(±386)	<0.0005

Hospital stay [days; mean (±SD)]	18.05(±7.83)	21.15(±12.43)	0.287
Survival [months; mean (±SD)]	26.34(±4.66)	18.44(±5.53)	<0.0005

Supplementary Table 3: The postoperative survival of Low and High mir patients

microRNA	Low mir patients [months; mean±SD]	High mir patients [months; mean±SD]	P value
mir124	21.1±6.11 (n=32)	25.9±5.21 (n=37)	<0.005
mir182	22.6±5.69 (n=26)	24.0±6.59 (n=43)	0.37
mir27b	21.1±5.38 (n=31)	25.7±5.93 (n=38)	0.002
mirlet7b	21.1±6.52 (n=34)	25.8±5.07 (n=35)	0.001
mir221	25.3±6.03 (n=36)	21.5±5.99 (n=33)	0.01
mir181a	25.4±5.61 (n=29)	22.1±6.38 (n=40)	0.02
mirs-expression	26.3±4.66 (n=44)	18.4±5.53 (n=25)	<0.00005

Supplementary Table 4: Primary & secondary antibodies for western blot

Protein	Source	Host	Dilution
CDK2	CST #2546	Rabbit	1:1000
CDK4	CST #12790	Rabbit	1:1000
CDK6	CST #3136	Mouse	1:1000
CyclinD1	CST #2926	Mouse	1:1000
CyclinD3	CST #2936	Mouse	1:1000
CyclinE1	CST #4129	Mouse	1:1000
P27	CST #3686	Rabbit	1:1000
P21	CST #2947	Rabbit	1:1000
P18	CST #2896	Mouse	1:1000
Drosha	Abcam ab183732	Rabbit	1:2000
DGCR8	Santa sc134567	Rabbit	1:1000
PCNA	CST #2586	Mouse	1:2000
P53	Abcam ab131442	Rabbit	1:500
β-actin	THETM A00702	Mouse	1:2000
Anti-rabbit IgG	CST #7074	Goat	1:4000
Anti-mouse IgG	CST #7076	Horse	1:4000

Supplementary Table 5: Potential target genes of miRNAs predicted by bioinformatic algorithms (http://www.microrna.org/microrna/home.do)

microRNA	target genes	microrna.org
hsa-mir-124	CDK2	http://www.microrna.org/microrna/getMrna.do?gene=1017&utr=8737&organism=9606#
	CDK4	http://www.microrna.org/microrna/getMrna.do?gene=1019&utr=9090&organism=9606
	CyclinD1	http://www.microrna.org/microrna/getMrna.do?gene=595&utr=16437&organism=9606
	CyclinE1	http://www.microrna.org/microrna/getMrna.do?gene=898&utr=7355&organism=9606#
hsa-mir-182	CDK2	http://www.microrna.org/microrna/getMrna.do?gene=1017&utr=8737&organism=9606#
	CyclinD1	http://www.microrna.org/microrna/getMrna.do?gene=595&utr=16437&organism=9606
hsa-mir-27b	CDK2	http://www.microrna.org/microrna/getMrna.do?gene=1017&utr=8737&organism=9606#
	CyclinD1	http://www.microrna.org/microrna/getMrna.do?gene=595&utr=16437&organism=9606
hsa-mir-let7b	CyclinD1	http://www.microrna.org/microrna/getMrna.do?gene=595&utr=16437&organism=9606
hsa-mir-221	P27	http://www.microrna.org/microrna/getMrna.do?gene=1027&utr=35641&organism=9606
hsa-mir-181a	P27	http://www.microrna.org/microrna/getMrna.do?gene=1027&utr=35641&organism=9606

Supplementary Table 6: Primers for quantificational real-time PCR

mir124-RT	GTCGTATCCAGTGCGTGTCGTGGAGTCGGCAATTGCACTGGATACGACGGCATT
mir182-RT	GTCGTATCCAGTGCGTGTCGTGGAGTCGGCAATTGCACTGGATACGACAGTGTGA
mir27b-RT	GTCGTATCCAGTGCGTGTCGTGGAGTCGGCAATTGCACTGGATACGACGCAGAA
mirlet7b-RT	GTCGTATCCAGTGCGTGTCGTGGAGTCGGCAATTGCACTGGATACGACAACCACA
mir211-RT	GTCGTATCCAGTGCGTGTCGTGGAGTCGGCAATTGCACTGGATACGACGAAACCC
mir181a-RT	GTCGTATCCAGTGCGTGTCGTGGAGTCGGCAATTGCACTGGATACGACGGTACAA
mir149-RT	GTCGTATCCAGTGCGTGTCGTGGAGTCGGCAATTGCACTGGATACGACGGGAGTG
mir206-RT	GTCGTATCCAGTGCGTGTCGTGGAGTCGGCAATTGCACTGGATACGACCCACAC
mir423-RT	GTCGTATCCAGTGCGTGTCGTGGAGTCGGCAATTGCACTGGATACGACACTGAGG
mir638-RT	GTCGTATCCAGTGCGTGTCGTGGAGTCGGCAATTGCACTGGATACGACAGGCCGCG
mir663-RT	GTCGTATCCAGTGCGTGTCGTGGAGTCGGCAATTGCACTGGATACGACGCGGTCC
mir762-RT	GTCGTATCCAGTGCGTGTCGTGGAGTCGGCAATTGCACTGGATACGACGCTCGGC
mir1246-RT	GTCGTATCCAGTGCGTGTCGTGGAGTCGGCAATTGCACTGGATACGACCCTGCT
mir1268-RT	GTCGTATCCAGTGCGTGTCGTGGAGTCGGCAATTGCACTGGATACGACCCCCCAC
U6-RT	CGCTTCACGAATTTGCGTGTCAT
mir124-F	GTAAGGCACGCGGTGAA
mir182-F	GCGTTTGGCAATGGTAGAAC
mir27b-F	GCTTCACAGTGGCTAAG
mirlet7b-F	GCTGAGGTAGTAGGTTG
mir211-F	GCAGCTACATTGTCTGCT
mir181a-F	GCACCATCGACCGTTGA
mir149-F	GTCTGGCTCCGTGTCTT
mir206-F	GGGTGGAATGTAAGGAAGT
mir423-F	TTTAGCTCGGTCTGAGGCC
mir638-F	TTATAAGGGATCGCGGGCGGGT
mir663-F	ATATATAAAGGCGGGGCGCCGCG
mir762-F	ATATATAAGGGGCTGGGGCCGGG
mir1246-F	CCCAATGGATTTTTGGAG
mir1268-F	AATTTCGGGCGTGGTGGTGG
mir-R	CGTATCCAGTGCGTGTCGTG
U6-F	GCTTCGGCAGCACATATACTAAAAT
U6-R	CGCTTCACGAATTTGCGTGTCAT
mir124-pri-F	CCATCTTCTACCCACC
mir124-pri-R	CATTCACCGCGTGCCTTA
mir182-pri-F	TTTGGCAATGGTAGAACTCAC
mir182-pri-R	AGAACAGCAGGTCCAGCAT
mir27b-pri-F	CACCGTCCCTTTATTTATGC
mir27b-pri-R	GGCAGAACTTAGCCACTGT
mirlet7b-pri-F	TGCCATCTCCCCTGTCC
mirlet7b-pri-R	GGAAGGCAGTAGGTTGTAGAT

mir221-pri-F	CCTGGCATACAATGTAGATTTCTG
mir221-pri-R	CACTGGTTTATACCTCCTG
mir181a-pri-F	ACCATTCAACGCTGTCGGT
mir181a-pri-R	CACAGTTCAACCCACC
CDK2-F	TCCAGGGCCTAGCTTTCTGC
CDK2-R	CCACAGGGTCACCACCTCAT
CDK4-F	CTCTCTAGCTTGCGGCCTGT
CDK4-R	CAAGGGAGACCCTCACGCC
CDK6-F	GGAGTGCCCACTGAAACCAT
CDK6-R	CGTGACGACCACTGAGGTTA
CycinD1-F	CCCGATGCCAACCTCCTCAA
CycinD1-R	CTGTTCCTCGCAGACCTCCA
CyclinD3-F	GAGCTGCTGTGTTGCGAAGG
CyclinD3-R	CGCTGCTCCTCACATACCTCC
CyclinE1-F	GCCGCAGTATCCCCAGCAAA
CyclinE1-R	TCGCACCACTGATACCCTGA
P27-F	TAATTGGGGCTCCGGCTAAC
P27-R	GAAGAATCGTCGGTTGCAGGT
P21-F	CAGAGGAGGCGCCATGT
P21-R	GGAAGGTAGAGCTTGGGCAG
P18-F	TAACTTGAGGGCCACCGAAC
P18-R	TAGGGTCCCTTGTTCACGGT
PCNA-F	AGTGGAGAACTTGGAAATGGAA
PCNA-R	GAAGAGAGTGGAGTGGCTTTTG
P53-F	TGCTCAAGACTGGCGCTAAA
P53-R	CAGTCTGGCTGCCAATCCA
Drosha-F	GCCCGAGAGCCTTTTATAGGT
Drosha-R	ATAAGCTCAGAGACTGCCTCA
DGCR8-F	GCGCCTCAGGTAGAAGAAGA
DGCR8-R	ATGCACAAGTCCGCTGAGAC
Dicer-F	GATGGTTCTCGAAGGCCCG
Dicer-R	GCTTCAAGCAGTTCAACCTGATA
EXP5-F	GCTGGTTGCATTGGGAAACC
EXP5-R	ACAAGAAAGGCCGAGGAAGG
RnaseA-F	GCAGATCCAGGCTTTTCTGGG
RnaseA-R	GGAATTTCTTGGCCCGGGAT
RnaseL-F	GGGAGAGCCGCTATAGGACG
RnaseL-R	GATCACCCACAGTGTTCTGGT
β-actin-F	TGGCATCCACGAAACTACCT
β-actin-R	ACGGAGTACTTGCGCTCAG

Supplementary Table 7: Primers for luciferase report vectors (WT and MUT)

mir124-CDK2	
WT1-F	TGGACTAGTTGGACTCAGGTG
WT1-R	CAAAGCTTGGTACGGCAAATC
MUT1-F	CTACTAGTCTTGGCCAGCCAA
MUT1-R	CAAAGCTTGGTACGGCAAATC
WT2-F	TCCACTAGTAGAGTTGGC
WT2-R	CGGGAAGCTTATAAAAC
MUT2-F	TCCACTAGTAGAGTTGGC
MUT2-R	GCGCAAGCTTCAAACTAAA
mir124-CDK4	
WT-F	GGTACTAGTCCATCTTTCTACAG
WT-R	GGCAAAGCTTAACAGAGGAAGAA
MUT-F	CCACTAGTCCTTTTGAGGCTTCT
MUT-R	GGCAAAGCTTAACAGAGGAAGAA
mir124-CyclinD1	
WT1-F	CAACTAGTTACACACACACA
WT1-R	CCCGAAGCTTTTTAAAGGAAG
MUT1-F	CCGCACTAGTACAGTTTATT
MUT1-R	CCCGAAGCTTTTTAAAGGAAG
WT2-F	GCGCACTAGTTTTAGTTTTCTC
WT2-R	GCCAAAGCTTGCAGAACCTGCC
MUT2-F	GACCACTAGTGACTTAATGTGA
MUT2-R	GCCAAAGCTTGCAGAACCTGCC
mir124-CyclinE1	
WT-F	GGTACTAGTTGACCTAAGGGA
WT-R	GCCAAGCTTACAAAACAGTTC
MUT-F	GGTACTAGTTGACCTAAGGGA
MUT-R	GGCAAGCTTATAATGTGGAGA
mir182-CDK2	
WT-F	CCGACTAGTTCTCTTCCTTTTAG
WT-R	CCCAAGCTTACTCTACTATTGGA
MUT-F	CACTAGTCCCATTTTCCTCTGAC
MUT-R	CCCAAGCTTACTCTACTATTGGA
mir182-CyclinD1	
WT-F	GCGCAACTAGTTGAAGTTTAG
WT-R	CAGAAGCTTTGTGTGTGTGTG
MUT-F	AAACTAGTGACCTCAGAGGTT
MUT-R	CAGAAGCTTTGTGTGTGTGTG
mir27b-CDK2	
WT-F	TACTAGTTAGGGTTTAGGCATC
WT-R	GGACAAGCTTGACAGAGACAAA

MUT-F	GCGCACTAGTTCTTTAAAAG
MUT-R	GGACAAGCTTGACAGAGACAAA
mir27b-CyclinD1	
WT1-F	TTACACTAGTGTAGCGTGCCCGT
WT1-R	GCGCAAGCTTCTCATAAACAGGT
MUT1-F	GCCAACTAGTGACAAACCATCCA
MUT1-R	GCGCAAGCTTCTCATAAACAGGT
WT2-F	AGAGACTAGTCTGTGTCCCTCTT
WT2-R	CCCGAAGCTTGAACAAATTCCAG
MUT2-F	ATACACTAGTTCTGATCGGGGGC
MUT2-R	CCCGAAGCTTGAACAAATTCCAG
WT3-F	AAAGACTAGTGGCAAGTGCACGG
WT3-R	TTACAAGCTTTATTTCTAGACTT
MUT3-F	ATATACTAGTCGGCGGGCGGCT
MUT3-R	TTACAAGCTTTATTTCTAGACTT
mirlet7b-CyclinD1	
WT-F	CTGCACTAGTCCAAAAAGGTTGC
WT-R	GCCGAAGCTTCACGCAAAGAATA
MUT-F	TTCAACTAGTCATGGTGGACCCA
MUT-R	GCCGAAGCTTCACGCAAAGAATA
mir221-P27	
WT-F	AGGCACTAGTAAATGATCTGCC
WT-R	GCATAAGCTTTCTCTGAACGGG
MUT-F	ATAAACTAGTGGGAAGGGAGGG
MUT-R	GCATAAGCTTTCTCTGAACGGG
mir181a-P27	
WT-F	CTCCAACTAGTTTGGGGCAAAAA
WT-R	GCGCAAGCTTTAATGTGAAGTT
MUT-F	GCGCACTAGTTTTGTTGACAAA
MUT-R	GCGCAAGCTTTAATGTGAAGTT
